# The associations of working hour characteristics with short sickness absence among part- and full-time retail workers

**DOI:** 10.5271/sjweh.3952

**Published:** 2021-04-27

**Authors:** Rahman Shiri, Tarja Hakola, Mikko Härmä, Annina Ropponen

**Affiliations:** Finnish Institute of Occupational Health, Helsinki, Finland

**Keywords:** night shift, quick return, risk factor, shift work schedule, sick leave

## Abstract

**Objective:**

This study aimed to determine the associations of working hour characteristics with short (1–3 days) sickness absence (SA) among retail workers.

**Methods:**

As part of “RetailHours-project”, 4046 employees of 338 Finnish retail stores were included. Registry-based data on working hour characteristics and short SA were utilized. A case-crossover design was used and the odds ratios (OR) were controlled for the clustering effect and working hour characteristics.

**Results:**

There were strong dose–response relationships between percent of short (<11 hours) shift intervals and short SA among part- and full-time workers, men and women, and younger and older workers. Compared to workers without short shift intervals, the risk of SA was 1.47 times [95% confidence interval (CI) 1.29–1.68] higher among workers who had short shift intervals <10% of work times, 2.39 times (95% CI 2.03–2.82) higher among workers who had 10–25% of work times, and 4.03 times (CI 2.34–6.93) higher among workers who had short shift intervals >25% of work times. Weekly working hours >40 hours were associated with SA among part-time workers [odds ratio (OR) 2.22, CI 1.65–2.98], women (OR 1.62, CI 1.27–2.07) and among workers <30 years of age (OR 1.68, CI 1.20–2.35) as well as among workers aged ≥30 years (OR 1.43, CI 1.07–1.92). Furthermore, working mainly night shifts was associated with SA among full-time workers (OR 2.41, 95% CI 0.99–5.86) and women (OR 1.72, CI 1.02–2.89).

**Conclusions:**

A short shift interval is an important risk factor for short SA. Improving intervals between shifts and shortening long weekly working hours could reduce the risk of short SA among retail workers.

Sickness absence (SA) is commonly used as an indicator for monitoring work-related health (1). Prospective cohort studies found an association between shift work and SA (2, 3). A systematic review of studies published up to April 2010 found an association between fixed evening shifts and SA among female healthcare workers but showed inconclusive evidence for rotating and night shifts (4). Since then, studies utilizing register-based data among hospital workers showed that long weekly working hours (5), long shifts (≥12 hours) (6, 7), night shifts (3, 5), 2- and 3-shift rotations (3), and short (<11 hours) interval between shifts (5, 8) increased the risk of short SA. Shift work that included night work also increased the risk of short SA among female-dominated occupations (2). Furthermore, lack of influence on working hours (9), evening work (10), night shift work (10, 11), 3-shift schedule (12), and shifts that lasted ≥12 hours (6) increased the risk of long-term SA.

Retail grocery stores and supermarkets provide vital services to the communities. Retail workers are exposed to physical workload factors such as forceful lifting, forceful pushing, pulling or carrying heavy loads, repetitive movements of the hands or wrists, working with arms above the shoulder level, and awkward and static postures (13–15). As a consequence, retail workers are at increased risk of developing musculoskeletal disorders such as neck or shoulder disorders, back disorders, tendinitis, and carpal tunnel syndrome (13–18). Around half of women working at grocery stores reported neck or shoulder complaint in the preceding 7 days and 34% reported elbow or hand complaints (17). Since musculoskeletal disorders, particularly back and shoulder disorders, are common causes of SA (19), retail workers are at risk of SA. Retail workers are also exposed to psychosocial risk factors such as high job strain (14, 17, 20). An earlier study showed that the association between night shift work and short SA among female-dominated occupations is not due to differences in psychosocial factors between day and night shift workers (2).

To date, little is known about the effects of occupational risk factors on musculoskeletal disorders and their associated disability among grocery store workers (18). Of these workers, only cashiers have mostly been studied (14, 15, 21). Among retail grocery store workers, work schedules more often are unpredictable and unstable, and most workers have little control over their shift work schedules (22). Grocery store workers with unstable and unpredictable work schedules reported poorer sleep quality, had more difficulty in falling asleep, woke more frequently during sleep, and felt tired more often than workers with more stable and predictable work schedules (22). To our knowledge, to date, no study has identified the predictors of SA related to working hour characteristics among retail workers. Similar to healthcare workers, retail workers are predominantly women (5, 22), however, they are somewhat younger and more often work as a part-time job than healthcare workers (5, 22). Furthermore, it is unclear whether age, sex, or type of work contact (part-time or full-time) play a role in the associations between working hour characteristics and short SA. Some previous studies found an increased risk of SA only among older shift workers (10) or among older employees working >40 hours/week (23). Also, a study showed that part-time workers are at higher risk of SA than full-time workers (24).

The aim of the present study was to explore the associations of working hour characteristics with short (1–3 days) SA among retail workers. Moreover, we determined whether the associations differ between part- and full-time workers, men and women, and younger and older workers.

## Methods

### Population

Data were gathered as part of the development of working hours in retail project (“RetailHours-project”) that consists of three regions in a chain of companies in the retail sector in Finland. The regions were the capital area of Finland (N >11 000 employees), Middle Finland (N >2700) and Northeast Finland (N >2200). In total, in this chain of companies, there were 900 outlets across Finland and the RetailHours-project included 450 (50%) of them. The RetailHours-project had in total working hour data from 16 728 employees from 6 March 2017 to 31 December 2019. We selected the final sample to include only those who were employed by the three regions (ie, we excluded the agency workers who were employed by other companies and paid on an hourly basis, N=1411) and those who did not have working hours according to the collective agreement of sales sector (N=3864 being employees of other service sectors such as hospitality). Furthermore, the population of the current study was restricted to employees who had at ≥1 short (1–3 days) SA (ie, the first incidence of short SA since 6 March 2017) and had data on working hour characteristics during eight weeks before the first short SA (N=4046, 911 men and 3083 women). Since the data comprised employer-owned employment information without access to diagnosis-specific SA, no ethical approval was required for the study.

### Outcome

We used 1–3 SA days as the outcome of the study. In Finland and the other Nordic countries, a SA of ≤3 days does not need a medical certificate. For each participant, data on starting and ending SA was collected.

### Characteristics of working hours

Data on the working hour characteristics during eight weeks before SA were collected. The payroll-based employer’s owned registry data of daily working hours were retrieved from the shift scheduling program Ortec Workforce Scheduling (Elli)-program. Information on the number of weekly working hours, type of shift (early morning, morning, day, evening, and night), length of work shift, and percentage and number of short (<11 hours) shift intervals (interval between two consecutive work shifts) was collected. Also, data included information on age, sex, and part-time and full-time work based on the employment contract. The data did not contain reasons for part-time work (ie, if part-time work was due to health, childcare, studies or else).

For each participant, data included information on starting and ending of each work shift. We defined day shift as a shift of ≥3 hours between 08:00 and 18:00 hours, morning shift as a shift between 03:00 and 18:00 hours, evening shift as a shift between 18:00 and 23:00 hours, and night shift as a shift between 23:00 and 06:00 hours as modified from Larsen et al (10) and Härmä et al (25–26) for retail work. An early morning shift starts before 06:00 hours and is not classified as a night shift. The classification of the timing of the shift was not mutually exclusive, but we gave highest priority to the night shifts, the second highest to the evening shifts and the lowest priority to the day shifts (26). Early morning and morning shifts did not overlap with other shifts. To distinguish different shifts, we defined a particular shift (eg, night shift) as working ≥50% of the work time during four weeks in that shift, and those who worked <50% of the time in a particular shift were group in a separate category of any shift <50%. Day shift has the lowest health risk (26), however, due to a small number of day workers in the current study, we compared night, evening, early morning and day shifts with morning shift. We also classified the length of shifts into short (<4 hours), medium (4–9 hours) and long (>9 hours). We defined a short interval between two shifts (quick return) as an interval <11 hours (8, 27). Lastly, long weekly working hours was defined as working >40 hours and very long weekly working hours as working >48 hours per week.

### Statistical analysis

A case–crossover design was used to compare the working hour characteristics of the four weeks preceding SA (exposure window) with those of the four weeks before the exposure window (control window). In case–crossover design, each participant serves as his or her own control. A conditional logistic regression model was used, and the odds ratios (OR) were controlled for the clustering effect of 338 retail stores, shift work, number of consecutive night shifts, weekly working hours >40 hours, the length of shifts, and percent of short shift intervals. We conducted stratified analyses to determine whether there were differences in the associations of working-hour characteristics with short SA between part- and full-time workers, men and women, and between younger and older workers. We used median to split age into two groups: (i) workers <30 years and (ii) workers aged ≥30 years. Stata, version 15 (StataCorp LP, College Station, Texas) was used for the analyses.

## Results

The study population worked at 338 retail stores of various sizes, including small grocery stores, supermarkets and hypermarkets. Of the participants, 77.2% were women, and 73.4% worked part-time and 26.6% full-time according to employment contract (table 1). Participants were aged 15–74 years. Nearly half were <30 years, and only 5% were ≥60 years. The mean age of the participants was 34.6 [standard deviation (SD) 13.2] years, with men 32.2 (SD 11.5) years and women 35.4 (SD 13.5) years. A majority of the participants (64.4%) worked in the capital area of Finland. On average, 48% of full-time employees and 14% of part-time employees worked >40 hours/week for ≥2 weeks per month.

**Table 1:** The characteristics of the study population (N=4046)

Characteristic	N	%
Sex		
Men	911	22.5
Women	3083	76.2
Missing	52	1.3
Age (years)		
15–19	187	4.6
20–29	1805	44.6
30–39	772	19.1
40–49	572	14.1
50–59	509	12.6
60–74	201	5.0
Work schedule		
Full-time	1078	26.6
Part-time	2968	73.4
Region		
Capital area of Finland	2607	64.4
Middle-Finland	707	17.5
North-East Finland	732	18.1

### Work characteristics of short sickness absence

#### All workers

Long weekly working hours, short shifts (<4 hours), percent and number of short shift intervals (<11 hours) during four weeks of the exposure window were associated with short SA, while types of shifts, number of consecutive night shifts, and long shifts (>9 hours) were not associated with short SA (table 2). After adjustment for clustering effect and confounders, the risk of short SA was 1.52 times [95% confidence interval (CI) 1.25–1.85] higher among employees who worked >40 hours/week for ≥3 weeks during four weeks of the exposure window. The risk of SA was 1.30 times (95% CI 1.09–1.56) higher among employees who worked >48 hours/week for ≥1 week. Working night shift ≥1 night in a month, and the percentage and number of weeks working night shifts in a month were not associated with short SA.

**Table 2:** Odds ratios (OR) for the associations between work characteristics and short sickness absence among all workers. Number of weeks working >40 hours per week and extended weekly working hours at least for a week were not included in the same model II. Percent and number of short shift intervals were not included in the same model II. [CI=confidence interval.]

Characteristic	Control window		Exposure window		Model I ^a^		Model II ^b^	
			
	N	%	N	%	OR	95% CI	OR	95% CI
Shift work (>50% of 4 weeks)								
Day	126	3.1	113	2.8	0.87	0.60–1.26	0.98	0.67–1.44
Early morning	966	23.9	939	23.2	**0.68**	**0.48–0.97**	0.68	0.46–1.02
Morning	222	5.5	192	4.8	1		1	
Evening	1379	34.1	1316	32.5	0.95	0.78–1.17	1.00	0.81–1.24
Night	180	4.4	187	4.6	1.26	0.81–1.96	1.54	0.95–2.51
Any shift (<50%)	1173	29.0	1299	32.1	1.19	0.99–1.43	1.17	0.97–1.41
Number of consecutive night shifts								
0	3273	80.9	3313	81.9	1		1	
1–2	319	7.9	293	7.2	0.83	0.66–1.04	0.80	0.63–1.02
3–4	55	1.4	56	1.4	0.87	0.49–1.53	0.81	0.48–1.40
5	134	3.3	133	3.3	0.83	0.59–1.17	0.85	0.59–1.21
≥6	265	6.5	251	6.2	0.71	0.47–1.07	0.74	0.47–1.18
Number of weeks working >40 hour/week for 4 weeks								
0	1976	48.8	1916	47.4	1			
1	1148	28.4	1188	29.3	1.10	0.98–1.22	1.07	0.96–1.20
2	651	16.1	611	15.1	1.02	0.87–1.19	1.00	0.85–1.18
>3	271	6.7	331	8.2	**1.35**	**1.12–1.64**	**1.52**	**1.25–1.85**
Extended weekly working hours at least for a week / 4 weeks								
<40	1976	48.9	1916	47.4	1		1	
40–48	1660	41.0	1664	41.1	1.07	0.96–1.19	1.05	0.94–1.18
>48	410	10.1	466	10.5	**1.25**	**1.04–1.50**	**1.30**	**1.09–1.56**
Length of shifts (hours)								
Medium (4–9)	2251	55.6	2319	57.3	1		1	
Short (<4)	564	13.9	521	12.9	**0.83**	**0.70–0.98**	**0.84**	**0.70–0.99**
Long (>9)	1027	25.4	1019	25.2	0.94	0.83–1.07	0.91	0.79–1.05
Short and long	204	5.1	187	4.6	0.83	0.65–1.07	0.79	0.61–1.01
Percent of short (<11 hours) shift intervals								
0	1134	28.0	823	20.3	1		1	
<10	1841	45.5	1774	43.8	**1.45**	**1.28–1.65**	**1.47**	**1.29–1.68**
10.1–25	1025	25.3	1382	34.2	**2.33**	**1.98–2.74**	**2.39**	**2.03–2.82**
>25	46	1.2	67	1.7	**3.97**	**2.31–6.81**	**4.03**	**2.34–6.93**
Number of short (<11 hours) shift intervals /4 weeks								
0–1	2178	53.8	1799	44.5	1		1	
2–4	1597	39.5	1838	45.4	**1.55**	**1.39–1.74**	**1.57**	**1.40–1.76**
5–11	262	6.5	398	9.8	**2.49**	**1.96–3.16**	**2.51**	**1.97–3.19**
>12	9	0.2	11	0.3	**3.71**	**1.36–10.14**	**4.34**	**1.37–13.69**

a Model I: Adjusted for clustering effect.

b Model II: Adjusted for clustering effect, shift work, number of consecutive night shifts, number of weeks working longer than 40 hours per week, length of shifts, and percent of short shift intervals.

After adjustment for clustering effect and confounders, short SA was lower among workers who had short shifts [odds ratio (OR) 0.84, 95% CI 0.70–0.99]. The strongest associations were found between percent and number of short shift intervals (<11 hours), and short SA. Compared with workers with no short shift intervals, the risk of SA was 1.47 times (95% CI 1.29–1.68) higher among workers who had short shift intervals ≤10% of time during four weeks of the exposure window, 2.39 times (95% CI 2.03–2.82) higher among workers who had short shift intervals 10–25% of time, and 4.03 times (95% CI 2.34–6.93) higher among workers who had short shift intervals >25% of time. The risk of short SA also increased with increasing in the number of short shift intervals. The risk was 1.57 times (95% CI 1.40–1.76) higher among workers who had 2–4 short shift intervals in four weeks, 2.51 times (95% CI 1.97–3.19) higher among those who had 5–11 short shift intervals, and 4.34 times (95% CI 1.37–13.69) higher among those who had ≥12 short shift intervals compared with workers who had 0 or 1 short shift interval in four weeks.

#### Full-time versus part-time workers

The risk of SA strongly increased with increasing in percent of short shift intervals during four weeks of the exposure window among both full- and part-time workers (table 3). Among full-time workers, the risk of short SA was 2.41 times (95% CI 0.99–5.86) higher when working mainly night shifts and 1.38 times (95% CI 1.00–1.91) higher when working any shift for <50% compared with morning shifts. The risk of SA was lower among full-time employees who worked either short shifts (OR 0.65, 95% CI 0.42–0.98) or a combination of short and long shifts (OR 0.61, 95% CI 0.37–0.99) than those who worked 4–9 hours shifts. Among part-time workers, the risk of SA was 2.22 times (95% CI 1.65–2.98) higher among employees who worked >40 hours/week for 3 or 4 weeks and 1.51 times (95% CI 1.19–1.92) higher among employees who worked >48 hours/week for at least one week during four weeks of the exposure window. The number of consecutive night shifts, working night shift at least once a month, and the percentage and number of weeks working night shifts in a month were not associated with short SA among both full-time and part-time workers.

**Table 3:** Odds ratios (OR) for the associations between work characteristics and short sickness absence among part-time and full-time workers. Number of weeks working >40 hours per week and extended weekly working hours at least for a week were not included in the same model. [CI=confidence interval.]

Characteristic	Full-time (N=1078)						Part-time (N=2968)					
	
	Control window		Exposure window		OR ^a^	95% CI	Control window		Exposure window		OR ^a^	95% CI
							
	N	%	N	%			N	%	N	%
Shift work (>50% of 4 weeks)												
Day	19	1.8	11	1.0	0.58	0.18–1.88	107	3.6	102	3.5	1.01	0.66–1.55
Early morning	90	8.3	76	44.6	0.52	0.23–1.13	132	4.5	116	3.9	0.74	0.44–1.26
Morning	495	45.9	481	7.1	1		471	15.9	458	15.4	1	
Evening	162	15.0	147	13.7	0.99	0.65–1.51	1217	41.0	1169	39.4	0.98	0.77–1.25
Night	45	4.2	50	4.6	**2.41**	**0.99–5.86**	135	4.6	137	4.6	1.35	0.82–2.25
Any shift (<50%)	267	24.8	313	29.0	**1.38**	**1.00–1.91**	906	30.5	986	33.2	1.11	0.88–1.40
Number of consecutive night shifts												
0	852	79.1	864	80.1	1		2421	81.6	2449	82.5	1	
1–2	91	8.4	88	8.2	0.78	0.49–1.23	228	7.7	205	6.9	0.79	0.62–1.01
3–4	13	1.2	9	0.8	0.48	0.13–1.71	42	1.4	47	1.6	0.96	0.53–1.72
5	38	3.5	34	3.2	0.79	0.31–2.08	96	3.2	99	3.3	0.88	0.57–1.34
≥6	84	7.8	83	7.7	0.99	0.40–2.42	181	6.1	168	5.7	0.69	0.41–1.16
Number of weeks working >40 hour/week in 4 weeks												
0	210	19.5	225	20.9	1		1766	59.5	1691	57.0	1	
1	339	31.5	347	32.2	1.00	0.78–1.28	809	27.2	841	28.3	1.09	0.96–1.25
2	340	31.5	317	29.4	0.90	0.69–1.19	311	10.5	294	9.9	1.02	0.84–1.25
>3	189	17.5	189	17.5	1.17	0.83–1.65	82	2.8	142	4.8	**2.22**	**1.65–2.98**
Extended weekly working hours at least for a week / 4 weeks												
<40	210	19.5	225	20.9	1		1766	59.5	1691	57.0	1	
40–48	630	58.4	613	56.9	0.95	0.75–1.21	1030	34.7	1051	35.4	1.08	0.95–1.23
>48	238	22.1	240	22.2	1.09	0.81–1.46	172	5.8	226	7.6	**1.51**	**1.19–1.92**
Length of shifts (hours)												
Medium (4–9)	505	46.8	540	50.1	1		1746	58.9	1779	59.9	1	
Short (<4)	130	12.1	116	10.7	**0.65**	**0.42–0.98**	434	14.6	405	13.7	0.88	0.72–1.07
Long (>9)	376	34.9	363	33.7	0.86	0.65–1.15	651	21.9	656	22.1	0.93	0.80–1.09
Short and long % of short (<11 hours) shift intervals	67	6.2	59	5.5	**0.61**	**0.37–0.99**	137	4.6	128	4.3	0.87	0.64–1.16
0	255	23.6	145	13.5	1	879	29.6	678	22.8	1		
<10	487	45.2	438	40.6	**1.83**	**1.38–2.41**	1354	45.6	1336	45.0	**1.40**	**1.21–1.62**
10.1–25	316	29.3	465	43.1	**3.73**	**2.70–5.15**	709	23.9	917	30.9	**2.06**	**1.74–2.44**
>25	20	1.9	30	2.8	**6.81**	**2.47–18.75**	26	0.9	37	1.3	**3.12**	**1.62–6.01**

a Adjusted for clustering effect, shift work, number of consecutive night shifts, number of weeks working longer than 40 hours per week, length of shifts, and percent of short shift intervals.

#### Men versus women

Among both men and women, the risk of short SA strongly increased with increasing in percent of short shift intervals (table 4). Among women, the risk of short SA was 1.72 times (95% CI 1.02–2.89) higher when working mainly night compared with morning shifts. The risk of SA was 1.62 times (95% CI 1.27–2.07) higher among women who worked >40 hours/week for 3 or 4 weeks and 1.25 times (95% CI 1.00–1.56) higher among women who worked >48 hours/week for at least a week during four weeks of the exposure window compared with women who did not work >40 hours/week at any time during the four weeks. Moreover, women who worked only short shifts had lower SA (OR 0.79, 95% CI 0.65–0.97) than women who worked medium length shifts (4–9 hours). Among men, those who worked >48 hours/week for at least a week during four weeks of the exposure window had 1.45 times (95% CI 1.03–2.05) higher risk of short SA than men who did not work >40 hours/week at any time during the four weeks. The types of shift work, and length of work shift were not associated with short SA among men (table 4). The number of consecutive night shifts, working night shift for at least once a month, and the percentage and number of weeks working night shifts in a month were not associated with short SA among both men and women.

**Table 4:** Odds ratios (OR) for the associations between work characteristics and short sickness absence among men and women. Number of weeks working >40 hours per week and extended weekly working hours at least for a week were not included in the same model. [CI=confidence interval.]

Characteristic	Men (N=911)						Women (N=3083)					
	
	Control window		Exposure window		OR ^a^	95% CI	Control window		Exposure window		OR ^a^	95% CI
							
	N	%	N	%		N	%	N	%
Shift work (>50% of 4 weeks)												
Day	24	2.6	21	2.3	1.23	0.51–2.95	99	3.2	87	2.8	0.86	0.58–1.29
Early morning	56	6.2	50	5.5	0.75	0.31–1.84	166	5.4	141	4.6	0.64	0.37–1.10
Morning	224	24.6	214	23.5	1	730	23.7	717	23.2	1	
Evening	301	33.0	303	33.3	1.28	0.78–2.10	1052	34.1	986	32.0	0.91	0.72–1.15
Night	67	7.4	64	7.0	1.27	0.58–2.78	111	3.6	122	4.0	**1.72**	**1.02–2.89**
Any shift (<50%)	239	26.2	259	28.4	1.30	0.87–1.94	925	30.0	1030	33.4	1.10	0.89–1.35
Number of consecutive night shifts												
0	665	73.0	684	75.1	1	2561	83.0	2579	83.7	1	
1–2	94	10.3	81	8.9	0.71	0.46–1.10	224	7.3	210	6.8	0.86	0.65–1.12
3–4	20	2.2	25	2.7	0.91	0.45–1.82	34	1.1	31	1.0	0.80	0.37–1.70
5	42	4.6	37	4.1	0.58	0.30–1.14	92	3.0	96	3.1	1.06	0.69–1.64
≥6	90	9.9	84	9.2	0.56	0.27–1.15	172	5.6	167	5.4	0.88	0.49–1.58
Number of weeks working >40 hours/week in 4 weeks												
0	439	48.2	432	47.4	1	1503	48.8	1443	46.8	1	
1	256	28.1	264	29.0	1.08	0.86–1.34	880	28.5	917	29.7	1.10	0.96–1.25
2	150	16.5	147	16.1	1.08	0.79–1.48	495	16.1	464	15.1	1.01	0.83–1.22
>3	66	7.2	68	7.5	1.24	0.81–1.88	205	6.6	259	8.4	**1.62**	**1.27–2.07**
Extended weekly working hours at least for a week / 4 weeks												
<40	439	48.2	432	47.4	1	1503	48.7	1443	46.8	1	
40–48	373	40.9	361	39.6	1.04	0.84–1.30	1270	41.2	1300	42.2	1.08	0.95–1.24
>48	99	10.9	118	13.0	**1.45**	**1.03–2.05**	310	10.1	340	11.0	**1.25**	**1.00–1.56**
Length of shifts (hours)												
Medium (4–9)	1734	56.2	1774	57.6	1	480	52.7	498	54.7	1	
Short (<4)	408	13.2	365	11.8	1.00	0.71–1.42	152	16.7	156	17.1	**0.79**	**0.65–0.97**
Long (>9)	786	25.5	796	25.8	0.84	0.64–1.10	232	25.5	218	23.9	0.95	0.81–1.11
Short and long	155	5.0	148	4.8	0.80	0.47–1.37	47	5.1	39	4.3	0.80	0.61–1.05
% of short (<11 hours) shift intervals												
0	276	30.3	215	23.6	1	843	27.3	573	18.6	1	
<10	400	43.9	406	44.5	**1.44**	**1.10–1.87**	1417	46.0	1356	44.0	**1.58**	**1.36–1.83**
10.1–25	222	24.4	273	30.0	**1.90**	**1.43–2.53**	791	25.7	1104	35.8	**2.74**	**2.27–3.30**
>25	13	1.4	17	1.9	**3.38**	**1.15–9.93**	32	1.0	50	1.6	**4.74**	**2.72–8.28**

a Adjusted for clustering effect, shift work, number of consecutive night shifts, number of weeks working >40 hours per week, length of shifts, and percent of short shift intervals.

#### Younger (<30 years) versus older workers ( ≥30 years)

Among both younger and older workers, the risk of short SA strongly increased with increasing percent of short shift intervals (table 5). In workers <30 years, the risk of SA was 1.68 times (95% CI 1.20–2.35) higher among those who worked >40 hours/week for 3–4 weeks and 1.35 times (95% CI 1.03–1.77) higher among employees who worked >48 hours/week for at least a week during four weeks of the exposure window. In workers aged ≥30 years, the risk of SA were 1.43 times (95% CI 1.07–1.92) higher among those who worked >40 hours/week for 3–4 weeks, and 1.39 times (95% CI 1.11–1.75) higher when they worked any shift for <50% compared with morning workers. The risk of SA was lower among employees who worked either short shifts (OR 0.75, 95% CI 0.57–0.98) or a combination of short and long shifts (OR 0.60, 95% CI 0.42–0.86). The number of consecutive night shifts, working night shift for at least once a month, and the percentage and number of weeks working night shifts in a month were not associated with short SA among both younger and older workers.

**Table 5:** Odds ratios (OR) for associations between work characteristics and short sickness absence among younger (<30 years) and older (>30 years) workers. Number of weeks working >40 per week and extended weekly working hours at least for a week were not included in the same model. [CI=confidence interval.]

Characteristic	<30 years (N=1992)						>30 years (2054)					
	
	Control window		Exposure window		OR ^a^	95% CI	Control window		Exposure window		OR ^a^	95% CI
							
	N	%	N	%		N	%	N	%
Shift work (>50% of 4 weeks)												
Day	54	2.7	53	2.7	0.91	0.52–1.61	72	3.5	60	2.9	1.02	0.58–1.79
Early morning	92	4.6	81	4.1	0.63	0.38–1.03	130	6.3	111	5.4	0.72	0.39–1.34
Morning	311	15.6	316	15.8	1	655	31.9	623	30.3	1	
Evening	849	42.7	819	41.1	0.87	0.65–1.17	530	25.8	497	24.2	1.12	0.84–1.48
Night	108	5.4	113	5.7	1.31	0.78–2.18	72	3.5	74	3.6	1.73	0.79–3.78
Any shift (<50%)	578	29.0	610	30.6	0.96	0.73–1.25	595	29.0	689	33.6	**1.39**	**1.11–1.75**
Number of consecutive night shifts												
0	1539	77.2	1555	78.1	1	1734	84.4	1758	85.6	1	
1–2	177	8.9	170	8.5	0.85	0.62–1.17	142	6.9	123	6.0	0.75	0.56–1.01
3–4	39	2.0	40	2.0	0.86	0.46–1.61	16	0.8	16	0.8	0.82	0.32–2.07
5	83	4.2	81	4.1	0.80	0.51–1.28	51	2.5	52	2.5	0.91	0.47–1.76
≥6	154	7.7	146	7.3	0.77	0.40–1.48	111	5.4	105	5.1	0.71	0.38–1.35
Number of weeks working >40 hours/week in 4 weeks												
0	1138	57.1	1093	54.9	1	838	40.8	823	40.1	1	
1	534	26.8	547	27.4	1.06	0.91–1.24	614	29.9	641	31.2	1.08	0.90–1.28
2	226	11.4	233	11.7	1.12	0.90–1.40	425	20.7	378	18.4	0.92	0.73–1.14
>3	94	4.7	119	6.0	**1.68**	**1.20–2.35**	177	8.6	212	10.3	**1.43**	**1.07–1.92**
Extended weekly working hours at least for a week / 4 weeks												
<40	1138	57.1	1093	54.9	1	838	40.8	823	40.1	1	
40–48	701	35.2	717	36.0	1.07	0.93–1.24	959	46.7	947	46.1	1.03	0.87–1.22
>48	153	7.7	182	9.1	**1.35**	**1.03–1.77**	257	12.5	284	13.8	1.27	0.98–1.65
Length of shifts (hours)												
Medium (4–9)	1113	55.9	1123	56.4	1	1138	55.4	1196	58.2	1	
Short (<4)	316	15.9	298	14.9	0.92	0.73–1.16	248	12.1	223	10.9	**0.75**	**0.57–0.98**
Long (>9)	461	23.1	468	23.5	0.97	0.79–1.19	566	27.5	551	26.8	0.88	0.74–1.04
Short and long	102	5.1	103	5.2	0.97	0.69–1.36	102	5.0	84	4.1	**0.60**	**0.42–0.86**
% of short (<11 hours) shift intervals												
0	616	30.9	469	23.5	1	518	25.2	354	17.2	1	
<10	885	44.4	876	44.0	**1.44**	**1.22–1.70**	956	46.5	898	43.7	**1.51**	**1.25–1.83**
10.1–25	471	23.7	625	31.4	**2.22**	**1.77–2.79**	554	27.0	757	36.9	**2.60**	**2.07–3.26**
>25	20	1.0	22	1.1	**2.19**	**1.01–4.76**	26	1.3	45	2.2	**6.15**	**2.89–13.08**

a Adjusted for clustering effect, shift work, number of consecutive night shifts, number of weeks working longer than 40 hours per week, length of shifts, and percent of short shift intervals.

## Discussion

The present study showed that among factors related to shift work in the retail sector, a short shift interval is the stronger risk factor for short SA. Moreover, long weekly working hours increased the risk of short SA among part-time workers and working mainly night shifts increased the risk among full-time workers.

We found a strong dose–response relationship between a short interval between shifts (quick return) and short SA. A dose–response relationship was found among both part- and full-time workers, both men and women, and among both younger and older workers. In line with the current study, an earlier study utilizing objectively measured working hour characteristics among Norwegian nurses found a dose–response relationship between quick returns (<11 hours of rest between shifts) and SA (8). Furthermore, another study (5) also utilizing register-based data on working-hour characteristics found a short interval between work shifts increases the risk of short SA among Finnish hospital workers. However, an intervention aiming to increase interval between shifts improved sleep duration, alertness and well-being at work, but had no beneficial effect on the number of SA days (28). However, that study (28) recruited only 75 nurses, and the study had low statistical power to determine the effect of increasing interval between evening and morning shifts on the occurrence of SA. Quick return has adverse effects on sleep duration and causes sleepiness and fatigue (29) and can lead to absence from work.

To date, the association between shift work and SA is still uncertain. Some previous studies found that night shifts increase the risk of SA (2, 3, 5, 10, 11), while other studies found that evening shifts (4, 10) or rotating 2- or 3-shift (3, 12, 30) increase the risk of SA. Moreover, a study found no association between night shifts and long-term SA (12). Fixed night shifts (3), shift work including nightwork (night shift, 3-shift work, or rostered work including nights) (2) and working over 75% of time as night shifts (11) were associated with SA. Earlier studies reported inconsistent results on the association between consecutive night shifts and SA (5, 10). Working consecutive night shifts was associated with SA among Danish (10) but not Finnish (10, 23) healthcare workers. In the current study, we also found no association between consecutive night shifts and SA. The association between working ≥50% of time as night shifts and SA was found only among full-time workers and women. Night shift work can reduce sleep duration and sleep quality (31) and can cause mild depressive symptoms (2), which lead to a higher rate of SA. However, further large prospective studies are needed to confirm a positive link between night shift and SA.

Earlier studies showed that the rate of short SA is more common among hospital workers with extended weekly working hours (5, 23). In line with an earlier study among healthcare workers (23), we found that weekly working hours >40 hours increase the risk of short SA among both younger and older workers. The current study adds to our knowledge that working >40 hours per week increases the risk of short SA among women and part-time workers, whereas working >48 hours per week increases the risk among men. A study among healthcare workers (7) found an increase in the risk of short SA by 0.7–1.0% per week after introduction of the policy of extending shift length from 8 to 12 hours. Reduced sleep duration in employees who work long weekly hours (32) or long shift may play a role in their increased risk of short SA. Extended weekly working hours may also cause more fatigue among part- compared to full-time workers.

The study had some strengths and limitations. The study recruited a relatively large and representative sample of retail workers. Registry-based data on working hour characteristics in the shift work and SA were utilized. Furthermore, a case–crossover design was used, and each participant served as his or her control, which controlled the observed risk differences for the confounding effects of individual factors. However, the participants might have changed their level of exposure to physical and psychosocial factors during the exposure window. As a limitation of the current study, no information on exposure to workload and psychosocial factors was collected. However, the lag was limited to a maximum of four weeks. A 4-week lag between control and exposure windows is more optimal than a shorter or longer lag. A 4-week lag is needed to observe changes to shift patterns and working hour characteristics, while it is a short period to observe any meaningful changes in exposure to physical workload factors among workers with the same tasks. An earlier case-crossover study among healthcare workers (5) found that a 1-week and a 3-month lags between control and exposure windows produce the results similar to a 4-week lag. Lastly, some differences in working hour characteristics between exposure and control windows might have happened because of seasonal variation.

### Concluding remarks

The findings of the current study suggest that of working hour characteristics, a short shift interval is the most important risk factor for short SA among retail workers and avoiding it could reduce the risk of short SA. Moreover, shortening long weekly working hours, particularly among women, part-time workers and those <30 years could reduce the risk of short SA.

### Funding source

The Finnish Work Environment Fund (Grant # 190124) supported this study.

### Conflict of interest

The authors declare no conflicts of interest.
